# Why Are Omics Technologies Important to Understanding the Role of Nutrition in Inflammatory Bowel Diseases?

**DOI:** 10.3390/ijms17101763

**Published:** 2016-10-21

**Authors:** Lynnette R. Ferguson, Matthew P. G. Barnett

**Affiliations:** 1 Discipline of Nutrition and Dietetics and Auckland Cancer Research Society, Faculty of Medical and Health Sciences, The University of Auckland, Private Bag 92019, Auckland 1142, New Zealand; 2 Food Nutrition & Health Team, Food & Bio-Based Products Group, AgResearch Limited, Palmerston North 4442, New Zealand; matthew.barnett@agresearch.co.nz

**Keywords:** Inflammatory bowel disease, transcriptomics, metabolomics, proteomics, microbiomics

## Abstract

For many years, there has been confusion about the role that nutrition plays in inflammatory bowel diseases (IBD). It is apparent that good dietary advice for one individual may prove inappropriate for another. As with many diseases, genome-wide association studies across large collaborative groups have been important in revealing the role of genetics in IBD, with more than 200 genes associated with susceptibility to the disease. These associations provide clues to explain the differences in nutrient requirements among individuals. In addition to genes directly involved in the control of inflammation, a number of the associated genes play roles in modulating the gut microbiota. Cell line models enable the generation of hypotheses as to how various bioactive dietary components might be especially beneficial for certain genetic groups. Animal models are necessary to mimic aspects of the complex aetiology of IBD, and provide an important link between tissue culture studies and human trials. Once we are sufficiently confident of our hypotheses, we can then take modified diets to an IBD population that is stratified according to genotype. Studies in IBD patients fed a Mediterranean-style diet have been important in validating our hypotheses and as a proof-of-principle for the application of these sensitive omics technologies to aiding in the control of IBD symptoms.

## 1. Introduction

Inflammatory bowel diseases (IBD), including Crohn’s disease (CD) and ulcerative colitis (UC), are immune-mediated disorders characterised by chronic and relapsing inflammation of the gastrointestinal tract. Although IBD were rarely observed a century ago, their prevalence has increased in recent years [1]. For example, an Australia/New Zealand survey in 2014 showed high and increasing rates of IBD, especially CD [2]. While there is a clear genetic susceptibility to the diseases [3,4,5,6], diet [7], environment [8], and the resident intestinal microbial population (the microbiota) [9] are also thought to play an important role in the onset and progression of the disease.

Nutrigenomics New Zealand was a research programme established in 2004, with the aim of utilising the burgeoning field of omics technologies in identifying and developing bioactive compounds, functional foods, and/or diets for human consumption [10,11,12]. The initial target of this project was IBD, including both CD and UC. Although these diseases were initially seen merely as providing a proof of principle, they have proved to be extremely relevant to a range of inflammatory disorders, and provide an excellent example of the application of new developments in systems biology to nutrition and food science [13,14,15]. The insights provided are appropriate not only to the maintenance of good health, but also to protection against disease symptomology and progression.

The role of nutrition in IBD has been confused for many years, because it is clear that good dietary advice for one individual with the disease may not be the most appropriate advice for others [16,17,18]. Our hypothesis was that the differences in the impact of nutritional factors between individuals relate to genetic differences. As was already mentioned, there is strong evidence of a genetic basis to IBD, and this has largely been revealed by genome-wide association studies (GWAS), many of them done by the International IBD Genetics Consortium (IIBDGC) [3,19]. It has become increasingly clear that large numbers of subjects are important to reveal the genetic basis of disease, and IBD has almost become a gold standard for international collaborations. It is also important that diet is accurately assessed if gene-diet interactions are to be evaluated [20,21,22].

**Figure 1:**
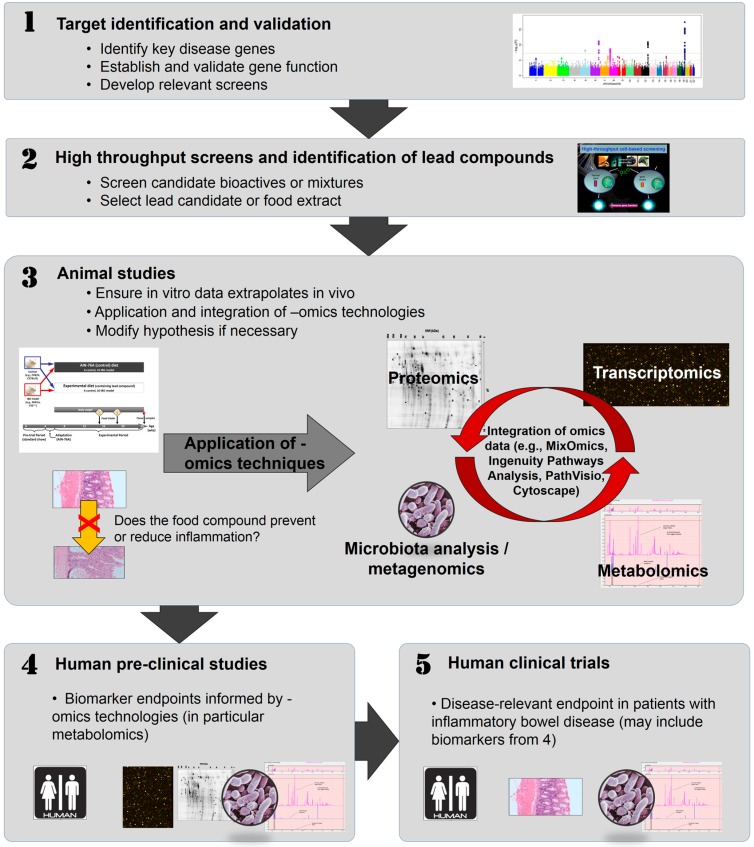
A potential sequence of in vitro to in vivo studies, including the application of omics technologies. This flow diagram shows a potential approach, working from in vitro through in vivo studies to human clinical trials, with the application of omics technologies at several stages. (**1**) There is increasing use of high-throughput techniques, such as single nucleotide polymorphism (SNP) chips, which enable genome-wide assessment to identify genetic factors which may be linked to a particular disease or health outcome. Relevant SNPs can then be incorporated into suitable in vitro assays (**2**) in which food compounds can be assessed for their abilities to interact with the SNP of interest and modulate its function. Effective food compounds can then be tested in appropriate animal models (**3**) which exhibit the relevant phenotype and/or have the SNP of interest (or one in a related gene). This can initially be used to establish if the food has an effect on phenotype and, subsequently a range of omics techniques can be applied, and data derived from them integrated, to better understand the mechanism by which a food may exert its effect. Food compounds which show efficacy both in vitro and in vivo may then be suitable as candidates for human studies, both pre-clinically (for example, to assess any possible biomarkers identified in the animal studies (**4**)) and, finally, clinically, to ascertain a clinically-relevant end-point, such as an improvement of IBD-associated symptoms (**5**). The Manhattan plot shown in (**1**) was originally published by Ikram, M.K.; et al. [36] and was obtained from Wikipedia (https://commons.wikimedia.org/wiki/File:Manhattan_Plot.png). This image file is licensed under the Creative Commons Attribution 2.5 Generic license.

Where genes important to an individual can be identified, this opens the possibility of exploiting the potential value of gene-specific bioactive food components in regulating the disease, using high throughput (HTP) screens. Again, many of the sensitive technologies provided by systems biology can be valuable in validating the potential effects of these compounds in animal or human studies. The omics approaches to foods enables an order of magnitude step in the understanding of their complexities and interactions with the host, and is sometime termed “foodomics” [23,24]. Foodomics has also been described as part of the host-microbiota-expososome interplay [25]. It highlights health benefits from nutrients and enables a detailed understanding of the metabolic consequences not only on the human body, but also on the gut microbiome. Transcriptomics, proteomics, and metabolic profiling are used, not only for screening food components, foods or diets for novel activity, but also in validating their effects in human populations [10,26,27]. A potential sequence of in vitro to in vivo studies is illustrated in Figure 1.

The rhizome of turmeric (*Curcuma longa* L., Zingiberaceae) can be crushed to form a yellow-orange powder. Not only has this powder long been used as a spice for cooking, it has also been used in traditional medicines in various countries, including India and China, for several centuries [28,29]. It is claimed to have beneficial effects against several inflammatory diseases, including IBD and cancer. The colour of turmeric is due to curcuminoids, including curcumin, which interact with a number of molecular targets [30]. Curcumin is claimed to act therapeutically in IBD, and is currently being developed for this purpose by the pharmaceutical industry [31,32]. Since various omics technologies have been used in the studies that played an important part in the proof of curcumin’s efficacy, curcumin will be used as an exemplar throughout the text.

## 2. Defining the Role of Genes in Inflammatory Bowel Disease (IBD)

IBD is almost a classic example of the importance of genetics in disease development. It was long recognised that there was a familial basis to the disease, and twin studies provided important information [33]. Specifically, it was shown that monozygotic twins had a higher concordance of disease risk than dizygotic twins. However, it was also apparent that environmental factors, such as smoking, played a significant role [33]. Early studies to understand the nature of the genetic component used association studies, but progress was slow. These studies compare the frequency of a chromosome variant, or a set of such markers, in unrelated patients with the disease, in comparison with a group of healthy controls. This enables the detection of markers which associate with disease risk. Linkage analysis considers DNA variations between a pair of linked markers, which will be associated with both markers. More specifically, linkage disequilibrium (LD) considers the extent to which an allele of one SNP is association with an allele of another SNP within the same population [34]. As with many other complex diseases, the genetic basis of IBD appears to reflect a combination of small variations in the DNA, typically in the form of single nucleotide polymorphisms (SNPs) or copy number variants (CNVs) that increase the susceptibility to disease, rather than directly causing it [35].

Technologies evolved, and genome-wide scanning using microsatellite markers identified shared regions of the chromosomes that were over-represented in diseased individuals, thereby improving the understanding of the disease. By 1996, the chromosome regions associated with IBD were identified as being on chromosome 16 [37], as well as 3, 7, and 12 [38]. Other chromosome regions followed fairly rapidly, and it was suggested that 1–9 chromosomal regions were associated with disease susceptibility [39]. In 2001, the first gene was unequivocally identified as associated with IBD risk—nucleotide oligomerisation domain 2 (*NOD2*) [40,41]—which mapped to chromosome 16. These authors reported three independent associations for CD, including a frameshift variant and two missense variants of the *NOD2* gene. This gene activates the nuclear transcription factor, nuclear factor NF-κB, and plays a role in the recognition of microbial pathogens.

Since this is a relatively rare complex of diseases, the importance of increased numbers of subjects for studies became apparent, leading to some important international collaborations. It had also become apparent that progress was relatively slow using the previous methods, and the field further progressed with the development of array technologies that scanned a number of genetic variants. A microarray involves the hybridisation of a target nucleic acid to a large set of probes attached to a solid support [42]. The original technologies used arrays of complementary DNA (cDNA), a double-stranded DNA, spotted onto glass microscope slides hybridised to a large set of oligonucleotide probes, to detect variations in a gene sequence. However, these methods rapidly evolved.

Association studies were increasingly being applied to population databases in GWAS [34]. Rather than considering individual candidate genes, GWAS uses a SNP chip that spans the genome, typically considering between 500,000 and 1,000,000 SNPs, and requiring far more complex statistical analyses. The Wellcome Trust Case Control Consortium used such an approach in a collaborative venture that included 17,000 subjects, both with and without diseases including IBD, and follow-up studies also examined high numbers of individuals from different populations [43]. While these studies initially identified approximately seven variants associated with CD, meta-analysis brought this number to 71 [35]. Such studies revealed hitherto unsuspected mechanisms, including autophagy, in disease pathogenesis. They also showed that a number of IBD loci are shared with other inflammatory diseases.

The Immunochip (more specifically, the Infinium^®^ ImmunoArray BeadChip) was developed by Illumina, covering just under 200,000 genetic polymorphisms and designed to span loci that had previously been associated with specific regions associated with major autoimmune and inflammatory diseases [44]. The cost was lower than more standard GWAS chips, because it was produced in very large numbers. This cost and specificity was part of the rationale that led the expansion of the International IBD Genetics Consortium (IIBDGC), and led to the multi-authored paper by Jostins et al. [19]. These authors performed a meta-analysis of the then-current GWAS studies of CD and UC, followed by extensive validation of significant findings. They used the Immunochip method to add data from an extra 14,763 CD cases, 10,920 UC cases, and 15,977 controls, leading to a combined total of more than 75,000 cases and controls. They found 71 new associations, leading to a total of 163 IBD loci that met statistical significance. It was also apparent that many IBD loci are important in other immune-mediated disorders, especially ankylosing spondylitis and psoriasis. The loci also overlapped with those associated with susceptibility to mycobacterial infection.

Subsequent work using the Immunochip has revealed specific variants associated with the diseases in certain specific populations. For example, Huang and co-workers identified particular SNPs that associated with African Americans, while Yang et al. described six additional susceptibility loci occurring in Korean populations [45,46]. However, in general, there is considerable commonality across populations [47].

Genetic analysis has also been important in enabling better predictions of disease location and pathogenesis. The study by Cleynen et al. included patients from 49 centres across 16 European countries, as well as North America and Australasia [3]. They considered genotype-phenotype associations seen with current clinical sub-phenotypes in 34,819 patients overall (19,713 CD, 14,683 UC), all genotyped on the Immunochip array. They found that three loci (*NOD2*, major histocompatability complex (*MHC*), and macrophage stimulating 1 (*MST1* 3p21)) associated with IBD sub-phenotypes, especially disease location. They found that the genetic risk score strongly distinguished colonic from ileal CD, and suggested that there is a continuum of disorders within IBD, much better explained by these three groups than by CD and UC, according to the current definitions. The location and its genetic components were shown to have major implications for changes in disease pathogenesis over time.

## 3. Defining the Phenotype in IBD

**Figure 2:**
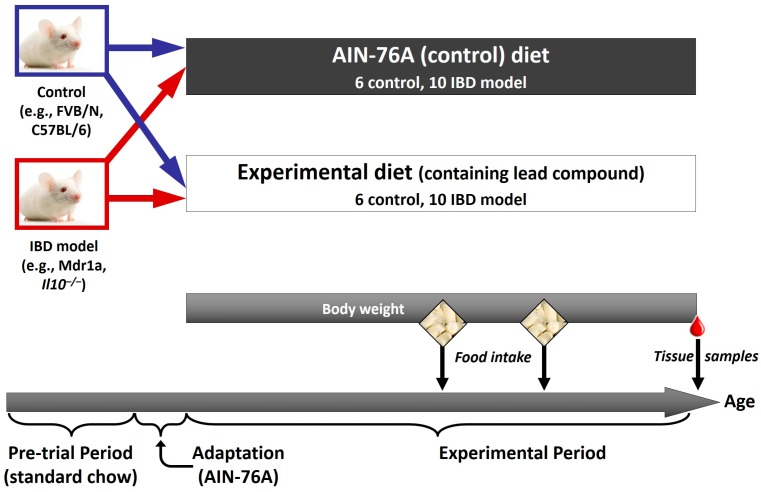
Experimental design for an in vivo study. An example of the design of an animal study in which the effect of a food or food compound on a phenotype, and a range of associated omics outcomes, can be assessed. In this case, a relevant model of IBD (such as the multidrug resistance 1 (Mdr1a) or interleukin-10 gene-deficient (*Il10^−/−^*) mouse) and its appropriate control is used. Both control and IBD model are fed either a control diet (such as the AIN-76A rodent diet), or the control diet supplemented with a food compound that has shown potential in an in vitro model. Body weight and food intake can be assessed during the experimental period, and at the end of the study a range of samples is collected. This would include intestinal samples to assess any effects of the compound on intestinal inflammation, as well as a range of less invasive samples (blood, urine, faeces) in which potential biomarkers of the intestinal phenotype may be present, which could be applicable to future human studies.

While genetics plays a major role in IBD characteristics and progression, the desire of personalising nutrition in this disease is that strategic dietary regulation may be able to modify disease symptomology. Important technologies are summarised below, and an illustration as to how these technologies have been applied to animal studies (for example, of curcumin) in IBD is provided in Figure 1, with an example of the animal study design shown in Figure 2.

### 3.1. Transcriptomics

The transcriptome is defined by the set of all messenger RNA (mRNA) molecules produced in one or a population of cells [48]. Thus, it provides a measure of the genes that are actively being expressed at a given time. The field of transcriptomics has been dominated by microarray solutions provided by companies such as Affymetrix, Agilent Technologies, and Illumina. However, RNA-Seq (defined as transcriptome profiling using next-generation sequencing (NGS)) is increasingly being utilised for such work. The stated advantages of RNA-Seq over microarrays include providing better detection of low abundance transcripts, and having a greater detection of a wide range of transcripts as compared with microarrays [49]. However, the cost is considerably greater than for microarrays at present, and it is not being as widely used as the older array-based technology.

Preliminary data from such analyses are often presented in the form of a heat map that visually displays the genes that are differentially regulated before and after a treatment, and the strength of the changes involved [50]. Having obtained the levels of differentially expressed mRNA probes, it becomes important to understand the functions and/or biological pathways which they are involved in. A number of authors have utilized software packages such as Ingenuity Pathways Analysis (IPA) for such analyses [50,51]. However, the cost of this may argue against its use in many studies. Free software, such as PathVisio, that visualizes data and enables pathway statistics, or Cytoscape, for integrating complex networks, have been used by a number of groups [52].

### 3.2. Epigenetics Analyses

The genome reveals the potential nature of cellular proteins, while the transcriptome shows which proteins are currently being transcribed through the formation of mRNA. Epigenetic events alter the expression of genes without altering the nature of the DNA code. Various important mechanisms include DNA methylation, histone modifications, and non-coding RNAs (ncRNA). The latter group represents the majority of such events in mammals, ranging from short ncRNAs including microRNAs, to long stretches of RNA (long ncRNA or lncRNA) [53]. Interestingly, there is increasing evidence for interactions between these two groups. Epigenetic mechanisms to regulate gene expression are critical in the cellular response to environmental stimuli, including diet. While hypothesis-based studies dominated earlier work, genome-wide analyses, e.g., the methylation specific amplification microarray (MSAM) consider variations across the genome [54]. NGS technologies are also increasingly used to study epigenetic changes.

### 3.3. Proteomics

Proteomics has been defined as “the analysis of the protein complement present in a cell, organ, or organism at any given time [55]”.

While the genome describes the potential cellular proteins, and the transcriptome gives a measure of which genes are being transcribed at a given time, neither of these methods provides information on the actual protein composition (“the proteome”). Since the protein, in general, represents the functional unit derived from genes and mRNA, it ultimately determines the phenotype and accurate assessment of the proteome is, therefore, essential. Proteins and/or peptides are generally separated out through gel electrophoresis or liquid chromatography, both of which can be either one- or two-dimensional, and are typically coupled with mass spectrometry. A comprehensive description of current technologies for a proteomics approach to the assessment of biological samples has been published relatively recently [55].

A commonly employed method to assess the proteome is 2D gel electrophoresis using difference gel electrophoresis (DIGE) technology, by which (for example) proteins differentially expressed during intestinal inflammation can be recognized, and subsequently identified using mass spectrometry technology (liquid chromatography-mass spectrometry (LCMS) or LC-MS/MS). Although multidimensional liquid chromatography-mass spectrometry (MDLC-MS) approaches are increasingly being applied for qualitative and quantitative proteome analysis (as reviewed: [56,57]), a combined 2D DIGE/MS approach remains an important and widely used technique. This approach can also be used to identify changes in the proteome when inflammation is reduced in response to dietary compounds, such as curcumin [51], and to, therefore, better understand the mechanisms by which inflammation is ameliorated.

### 3.4. Metabolomics

Metabolomics is increasingly being used to study both the nature of diseases such as IBD, and the implication of variations of nutrition on disease characteristics. Formally, this field has been defined as the “systematic study of the unique chemical fingerprints that specific cellular processes leave behind” [58]. The ultimate goal is to understand the effects of dietary components on metabolic regulation and consequent implications for human health. This is complicated by the fact that the metabolites that make up the profile of a given sample may result from at least three different sets of signals: (1) dietary compounds (nutrients and bioactives); (2) xenobiotics from environmental sources which are absorbed and metabolized; and (3) metabolic signals produced by the large-bowel microflora.

Metabolomics data can be used to identify overall patterns or effects, for example by applying methods such as partial least squares discriminate analysis to assess how the metabolite profile of an individual compares to those of other individuals. Such an approach may be applied to better diagnose a health outcome, such as irritable bowel syndrome [59], or to distinguish between different dietary treatments, outcomes which cannot be achieved with conventional techniques. Metabolomics can also be used to identify specific metabolic changes in response to an intervention, such as a diet, although the success of this approach depends on libraries of small molecules being developed to facilitate metabolite identification. A major technical challenge faced by metabolomics is the wide variety of chemical and physical properties of the metabolites, ranging from highly polar through to non-polar compounds.

One critical aspect of metabolomics which has driven many of the recent advances is the potential for collection of relatively non-invasive samples, such as faecal, urine, blood, buccal swabs, or breath volatiles. Based on data derived from appropriate animal models, such samples can enable the accurate assessment of important outcomes within a target tissue (such as the gastrointestinal (GI) tract) based on their metabolite profile. Technologies for metabolomics research broadly fall into two categories, being either nuclear magnetic resonance (NMR)-spectroscopy or mass spectrometry-based. However, within these are a range of sub-categories, reflecting the varied nature of the metabolites within a sample. For example, MS-based technologies cover the spectrum from polar (hydrophilic interaction chromatography (HILIC) for compounds such as amino acids, organic acids, di/trisaccharides) through semi-polar (C18, e.g., flavonoids) to non-polar (C1, e.g., phospholipids, triacylglycerols and diacylglycerols).

### 3.5. Metagenomics

It has become increasingly clear that the intestinal microbiota (bacteria inhabiting the gastrointestinal tract) play an essential role in human health. Indeed, some authors have likened the microbiota to an organ in its own right [60]. The colonic microbiota plays an even more critical role in IBD pathogenesis. Metagenomics is the current technology which describes the microbial genomic composition of the GI tract, usually measured from a faecal sample [61]. Changes in the composition of these bacterial populations, and/or of their collective genome, can have important implications for disease susceptibility, including intestinal inflammation [62,63].

Early studies on these populations required culture-based techniques, using anaerobic conditions. More recently, culture-independent techniques including NGS have provided important advances in understanding the diversity of the microbiota. The species composition can be measured using 16s rRNA sequencing, while comprehensive sequencing of the collective genome enables an understanding of the functional capacity of the microbiota. In addition, RNA-seq approaches allow a measurement of the expression of microbial genes to be measured. Hobbs et al., [61] developed a statistical method to characterise transcriptional regulatory networks from metagenomics data. Such approaches allow an assessment of the complex interplay between host and microbial genes, and their impact on the health of the host.

## 4. In Vitro Approaches to Identifying Bioactive Food Components Likely to Benefit IBD

Once the nature of genetic variations related to disease susceptibility are unequivocally established, this information can be used to design a cell line or other high-throughput screen that reflects the changes in cellular function associated with the disease. Such an approach can mimic the genotype, thereby allowing either food fractions or known dietary components to be considered for their ability to overcome the effect of the defective gene [64,65].

The genotype-specific NOD2 assay developed by Philpott and co-workers [66] provides an example of a genotype-specific assay. As already indicated, a functional *NOD2* gene activates the nuclear transcription factor NF-κB, and plays an important role in the recognition of microbial pathogens. The *NOD2* gene is also known as caspase-activated recruitment domain 15 (*CARD15*), and it encodes an intracellular receptor for the bacterial component muramyl dipeptide (MDP) [67]. An important component in normal cellular signalling is the production of the inflammatory cytokine tumour necrosis factor-α (TNF-α), which has several functions in the inflammatory process that is part of a general immune response to tissue damage and infection. It is central to the pathogenesis of CD because it is produced in excess during chronic inflammation. Part of this process involves stimulation of a signal transduction pathway activating NF-κB [68]. Philpott et al. developed a screen to test for food extracts with the potential to reduce inflammation mediated by this event [66]. The *NOD2* 3020insC polymorphism is a relatively common and frequently studied genetic variant in CD [69]. It is a frameshift mutation that leads to the formation of a premature stop codon, which prevents the ability of the cell to respond to MDP and, subsequently, to activate the transcription factor NF-κB. This assay thus models the *NOD2* 3020insC genotype and considers whether any food compound or extract has the ability to restore the normal signalling activity of cells carrying this variant, in comparison to that of cells carrying the wild-type gene.

A pUNO-hNOD2a vector was used in transfections as the wild-type *NOD2* reference vector. The 3020insC *NOD2* variant (subsequently designated pUNO-hNOD2a 3020insC) was generated from this by site-directed mutagenesis, using the Gene Tailor Site-Directed Mutagenesis System. For the experiments, the human embryonic kidney cell line 293 T (HEK293T) was grown according to standard protocols. These cells were transfected using Lipofectamine 2000 with a galactosidase vector (transfection control), pNifty2-SEAP vector (as the NF-κB reporter) and either the wild-type (to give a *NOD2* wild-type cell line) or constructed vector (to give the *NOD2* mutant cell line), or no other vector (the control cell line). Cells were allowed to settle into multi-well dishes and grown for a time before the addition of standard compounds or food extracts over a serial dilution range. After a further 24 h incubation, cells were stimulated by the addition of MDP or had an equivalent volume of media added. After further incubation, an aliquot of the cell supernatant was transferred to fresh plates containing a Quanti-Blue reagent for the detection of secreted alkaline phosphatase (SEAP) production, or treated with WST-1 reagent to measure cell viability. Absorbances were read at 635 nm (Quanti-Blue) and 450 nm (WST-1), and SEAP production was normalised against transfection efficiency. To compensate for any possible toxicity of extracts, SEAP production was also normalized against cell viability.

An example more relevant to curcumin, but using a similar rationale, was studied by McCann and co-workers [30]. Among various molecular pathways involved in susceptibility to IBD are those involved in intestinal epithelial barrier function and immune response [70]. The solute carrier family 22 member 4 (*SLC22A4*) gene is important for barrier function, encoding an organic cation transporter protein involved in the movement of proteins across the membrane of epithelial cells. McCann et al. considered the risk variant (rs1050152 at position 503F) which has a higher transport activity compared with the wild-type gene, leading to inappropriate transport of organic cations across the intestinal epithelial barrier [71]. As their example of a mutant that affected immune response, they utilised a variant in the gene encoding interleukin-10 (*IL10*). This is an immune-suppressive cytokine that reduces excessive inflammatory response, thereby creating an imbalance between pro- and anti-inflammatory mechanisms [72]. The rs1800896 SNP in this gene at position −1082 (the −1082A variant) reduces *IL10* transcription and cytokine production in IBD [73]. Thus, these authors considered the effects of an extract of turmeric and factions of this extract to overcome the functional effects of the two IBD-associated genetic variants—solute carrier protein 22 A4 (*SLC22A4*, rs1050152) and interleukin-10 (*IL10*, rs1800896). They developed an assay using HEK293 cells to examine the in vitro capacity of a turmeric extract, and fractions thereof, to affect the functionality of these two gene variants [30]. In parallel, they measured the curcumin content of the extract and fractions using high pressure liquid chromatography (HPLC). They found that an extract of turmeric beneficially affected the gene variants associated with IBD, by a reduction of inappropriate epithelial cell transport (*SLC22A4*, 503F) or through an increase in gene promoter activity of the IL10 anti-inflammatory cytokine (*IL10*, −1082A). The effect of turmeric on *IL10* was linked with its curcumin content. A similar effect was seen for several chromatographically separated fractions of turmeric.

In the progressive development of curcumin as a pharmaceutical solution to IBD, the low bioavailability of the purified compound has been recognised as important. This is largely caused by its low solubility and stability in the digestive tract. Several different formulations have been developed to overcome this problem, including a solid lipid particle formulation (Curcumin SLCP, Longvida) [74]. These authors considered the anti-inflammatory effects of such a formulation in lipopolysaccharide (LPS)-stimulated RAW 264.7 cultured murine macrophages. They utilised transient transfection technologies with an NF-κB reporter construct to show that this derived compound could inhibit the transcriptional activity of nuclear factor NF-κB in this cell line, and that it was more efficient in this process than the original material.

If a tentative bioactive food compound has been previously identified and seems likely to have potential in IBD, then several of the omics technologies can be applied to test the range of potential mechanisms of action. While effective activation of the immune response is critical to maintaining human health by responding to invading pathogens or other sorts of potentially deleterious injury, over-activation of the immune response leads to inflammation. Activation of the inflammasome is mediated by the innate immune system, and plays an important role in these responses [75]. Several pattern recognition receptors (PRRs) are components of the inflammasome complex, including the nucleotide-binding domain, leucine-rich repeat containing proteins (NLRs or NOD-like receptors) in both mice and humans. The Human Inflammasomes RT Profiler™ PCR Array measures the expression of 84 of the genes associated with this process, which have been linked to various diseases, such as IBD. Thus, although Miller and co-workers had planned their inflammasome array studies on curcumin to provide a comment on its potential in malignant mesothelioma, the data are also relevant to IBD [75]. These authors used mouse and human malignant mesothelioma cell models, in in vitro studies. Curcumin significantly down-regulated levels of expression of various genes involved in inflammation, including *NF-κB*, toll-like receptor (*TLR*), and interleukin 1 (*IL-1β*).

Proteomics was applied to studying the molecular targets of curcumin on a range of gastric cancer cell lines [76]. As well as defining dose response curves and demonstrating that curcumin caused apoptosis, they used two-dimensional gel electrophoresis (2-DE) and matrix-assisted laser desorption/ionization-time of flight (MALDI-TOF-TOF) mass spectrometry to study the target proteins. Of the proteins which showed more than a 1.5-fold change in curcumin-treated cells compared to untreated controls, 33 proteins were up-regulated and 42 proteins down-regulated by the treatment. Of relevance to IBD were 16% of proteins involved in metabolism and 2% in immune response. A similar study was carried out in the human breast cancer cell line MCF-7. Proteomic identification of differentially-expressed proteins in curcumin-treated MCF-7 cells identified 12 such proteins, although these genes did not seem to relate specifically to inflammatory diseases [77].

In vitro studies have also been informative on the likely effects of curcumin on the gut microbiome. Lou and coworkers [78] obtained faecal samples from a healthy male volunteer who had avoided alcohol or foods rich in curcumin for the previous 48 h. Following homogenisation of the sample, the supernatant was cultivated in an anaerobic chamber, divided into two and curcumin added to one of the subcultures, followed by overnight incubation. Ultra-performance liquid chromatography/quadrupole (UPLC-Q)-TOF MS enabled identification of the curcumin metabolites that were produced by the intestinal bacteria in the culture. They revealed novel metabolic pathways of curcumin in its interactions with the microbiome.

## 5. Animal Studies to Confirm the Role of Bioactive Components Likely to Benefit IBD

As described in the previous section, in vitro studies can provide highly relevant data via the application of omics techniques in a variety of contexts, including IBD. However, to date, there is no in vitro model which can adequately model the level of complexity of a biological system such as the GI tract. It is necessary to clearly establish safety and efficacy in a model before considering a human intervention, and animal models are currently the only viable option to achieve this. Thus, although human clinical outcomes are critical to establish the impact of any proposed nutritional intervention, there is still a place for appropriate animal models, for three key reasons.

As already mentioned, the GI tract is a highly complex environment in which food compounds, host cells, and the intestinal microbiota interact, and this cannot yet be adequately modelled in vitro. Animal models enable such modelling, which is important to better understand the mechanisms by which any impact of an intervention may occur. They also provide the ability to measure changes in the relevant tissues (in this case, intestinal tissue) and correlate these with peripheral changes (e.g., urine, blood) which have the potential to be used as biomarkers in a minimally-invasive manner for any subsequent human study; for example, to monitor symptoms and, therefore, assess the efficacy, or otherwise, of any intervention. This is of particular importance in the case of metabolomics, which is increasingly being applied to matrices, such as plasma and urine. It is also necessary to compare and correlate microbiota from the GI tract with those in faecal samples, so that subsequent analysis of faecal samples from human intervention studies can be used with a reasonable degree of confidence as a proxy for what is occurring in the GI tract.

Finally, it is important to establish robust use of omics techniques before applying them to human samples, and animal models provide the opportunity to do just that.

As an example, we describe some work of our own, and of others, on the potential beneficial effect of curcumin on intestinal inflammation. This includes what may be direct effects of curcumin on intestinal inflammation, as well as some which more likely occur through modulation of the intestinal microbiota.

Within the Nutrigenomics New Zealand programme, two mouse models of IBD were primarily utilized: the interleukin-10 gene-deficient (*Il10^−/−^*) mouse [79,80,81], and the multiple drug resistance (Mdr1a) mouse [82,83,84]. Both of these models exhibit symptoms similar to those seen in human IBD, resulting from the disruption of either the *Il10* gene (which encodes the anti-inflammatory cytokine interleukin-10) or the *Abcb1a* gene (also known as Mdr1a, which encodes the membrane drug-efflux pump p-glycoprotein 3), and both have been used to investigate the effects of a range of foods or food components on inflammation.

We have used the Mdr1a model to assess the effects of polyphenols, including those from green tea [82] and, of particular relevance for the current discussion, curcumin [51,84]. Initially using a transcriptomics approach, we showed that curcumin’s beneficial effect on colon inflammation may occur through up-regulation of xenobiotic metabolism and down-regulation of immune response pathways, in particular those mediated by the pregnane X receptor (Pxr) and peroxisome proliferator-activated receptor α (Pparα) activation of retinoid X receptor (Rxr). We also provided evidence that curcumin may down-regulate genes involved in oxidative stress and fibrogenesis pathways [84]. Subsequent use of proteomics analysis, and integration of gene and protein data, demonstrated that curcumin may regulate the actin cytoskeleton and thereby enhance the integrity of the intestinal barrier (a potentially important factor in inflammation) via α-catenin [51].

Other research suggests that the beneficial effects of curcumin for colitis may be as a result of maintaining a more diverse colonic microbial ecology [85]. A reduction in the diversity of the intestinal microbiota has been associated with intestinal inflammation in animal studies, including mice [86] and pigs [87], and in human studies [88] and, therefore, the maintenance of diversity represents a plausible mechanism by which curcumin may exert its effect. Systems biology approaches on IBD have usefully begun to focus on gut microbial metabolism [89].

## 6. Human Studies

As we have described above, a wide range of in vitro and animal studies support the conclusion that curcumin is likely to be effective both in preventing inflammation, and in suppressing the negative effects of inflammation once the symptoms have begun [90]. Continuous oxidative damage can initiate the inflammatory signalling cascade in IBD. In this context, curcumin acts to scavenge free radicals and also up-regulate various antioxidant enzymes. It also alters a number of key signalling pathways, for example inhibiting cyclooxygenases 1 and 2 (COX-1 and COX-2), tumour necrosis factor-α (TNF-α), and the transcription factor NF-κB. In addition, it has been demonstrated as safe to be co-administered with conventional therapy, albeit in relatively small human trials to date, and is being actively developed as both a chemopreventive and pharmaceutical agent for IBD [91,92]. As Aggawal and co-workers point out, its TNF-α inhibition alone justifies the cost of this development, since current TNF-α inhibitors are used not only for IBD, but also for osteoarthritis, psoriasis, and ankylosis, at a cost of $15,000–$20,000 per person per year [31].

Despite the promise in other study approaches, curcumin has given variable results in human trials [93]. Indeed, to date, convincing data for curcumin alone was only available for two studies, comprising data for 99 patients. However, in combination with mainstream therapy, curcumin has significantly improved patient symptoms or allowed for a drop in dosage of corticosteroids. For example, Lang et al. showed that curcumin, in combination with mesalamine, induced remission of ulcerative colitis symptoms after four weeks of treatment [94]. Clinical remission was achieved by no patients in the placebo-alone group in this time, but by more than half of the patients also receiving curcumin. Most of the authors of these studies have concluded that larger clinical trials may need to be conducted. Additionally, we suggest that patient stratification according to genotype may be effective and could be considered for future work.

As with many other natural products, although curcumin shows some efficacy in vivo, it has a limited shelf-life and is not absorbed efficiently. A number of novel formulations have been developed to overcome these problems. Kitture and co-workers used another natural product, *Aloe vera*, to synthesise a template in which to immobilise the curcumin [95]. In antioxidant assays, the curcumin-loaded template showed superior properties to either the curcumin or *Aloe vera* alone. This loaded template also facilitated the efficient release of curcumin within five hours, quicker than could occur with curcumin alone. Closer to clinical applications is the use of nanoparticles to facilitate the colonic delivery of curcumin [32,96]. These particles have been found to accumulate preferentially in inflamed regions, and can, potentially, aid delivery to the areas that will most benefit from the compound. Various chemical structures have been compared in order to optimise the final material which goes into large-scale clinical trials.

## 7. Conclusions

The so-call omics approaches to studying disease are relatively recent, with increasingly sensitive and cost-effective technologies emerging over the past 10–15 years. IBD provide an excellent example where there have been order of magnitude changes in understanding the nature of the diseases, and the way in which natural (or other) therapies may interact in the natural course of the disease. Fundamental to the understanding of cellular mechanisms have been genomic studies, considering not only the human genes, but also the gut microbiome [89]. Transcriptomics, proteomics, and metabolomics are among many of the sensitive technologies that not only help to explain the nature of the disease, but enable short-term studies with highly sensitive endpoints to test new therapeutic strategies. We have used curcumin as an example for which several of these technologies have been applied to date. This example demonstrates that understanding the nature of the disease targets enables potential consideration of an increasing number of natural products, or more targeted therapies, that provide significant hope for better control of disease in future generations.
